# Supplementary material for: Prevalence and clinical profile of glaucoma patients in rural Nigeria—A hospital based study
Source: PLoS One. 2021 Dec 2;16(12):e0260965. doi: 10.1371/journal.pone.0260965 (PMC8639068; doi:10.1371/journal.pone.0260965)

PLOS ONE

Prevalence and clinical profile of glaucoma patients in rural Nigeria - A hospital based study

--Manuscript Draft--

**Manuscript Number:**	PONE-D-21-21579
**Article Type:**	Research Article
**Full Title:**	Prevalence and clinical profile of glaucoma patients in rural Nigeria - A hospital based study
**Short Title:**	Sociodemographic Characteristics of Glaucoma patients in rural Nigeria
**Corresponding Author:**	Uchechukwu Levi Osuagwu, Ph.D<br>Western Sydney University - Campbelltown Campus<br>Campbelltown, South Western Sydney, New South Wales AUSTRALIA
**Keywords:**	Primary Open angle glaucoma; Nigeria; Blindness; Intraocular pressure; Visual field defect; Normal tension glaucoma
**Abstract:**	Purpose: To determine the prevalence and clinical presentation of participants with glaucoma attending a public eye care facility in Nigeria<br>Method: Hospital based retrospective study of glaucoma participants aged 50 years and above seen over a 5-year period. Descriptive statistics summarized the demographic, clinical characteristics and treatment of the participants and determined the association of variables with gender and age. Prevalence of the glaucoma by type, and their 95% confidence intervals (CI) were also calculated.<br>Result: Of the 5482 case files that were reviewed, 995 (18.15%, 95% CI 17.15 - 19.19%) had glaucoma particularly primary open angle glaucoma (11.55%, 95%CI 10.73 – 12.42%) and were mostly females (564, 56.7%) aged 69 ± 12 years (range, 50 - 103 years). In contrast to other glaucoma types, the prevalence of primary angle closure glaucoma (3.68, 95%CI 3.22-4.22) increased by 15% over 5 years. The mean intraocular pressure ranged from 15 – 50 mmHg but higher in females than males (27.8 ± 6.1mmHg versus 26.6 ± 6.0 mmHg, $p<0.05$) who had comparable VA (0.58 ± 0.4 Log MAR) and cup-disc ratios ($p>0.05$). On presentation, the glaucoma hemi field test (GHFT) was outside the normal limits in 45.5% and 54.5% of males and females, respectively. The type of visual field defect was associated with glaucoma type ($P = 0.047$). Arcuate scotoma was most common (35.5%) across glaucoma types, paracentral scotoma more common in Secondary glaucoma while Seidel scotoma was highest in NTG (19.3%). Beta-blocker was the mainstay of management (42.2%) but more likely to be prescribed to males while more females received carbonic anhydrase inhibitors.<br>Conclusions: The high prevalence of glaucoma in older people remains a public health problem in Nigeria. The fact that about half of the participants presented with visual field defect suggests there is a need for public health messages to emphasize on early glaucoma screening, detection and management.
**Order of Authors:**	Ngozika E Ezinne
	Chukwuebuka S Ojukwu
	Kingsley K Ekemiri
	Obinna F Akano
	Edgar Ekure
	Uchechukwu Levi Osuagwu, Ph.D
**Additional Information:**	
**Question**	**Response**
**Financial Disclosure**<br><br>Enter a financial disclosure statement that	Not applicable

Powered by Editorial Manager® and ProduXion Manager® from Aries Systems Corporation

describes the sources of funding for the work included in this submission. Review the submission guidelines for detailed requirements. View published research articles from *PLOS ONE* for specific examples.<br><br>This statement is required for submission and **will appear in the published article** if the submission is accepted. Please make sure it is accurate.<br><br>**Unfunded studies**<br>Enter: *The author(s) received no specific funding for this work.*<br><br>**Funded studies**<br>Enter a statement with the following details:<br>• Initials of the authors who received each award<br>• Grant numbers awarded to each author<br>• The full name of each funder<br>• URL of each funder website<br>• Did the sponsors or funders play any role in the study design, data collection and analysis, decision to publish, or preparation of the manuscript?<br>• **NO** - Include this sentence at the end of your statement: *The funders had no role in study design, data collection and analysis, decision to publish, or preparation of the manuscript.*<br>• **YES** - Specify the role(s) played.<br><br>* typeset	
**Competing Interests**<br><br>Use the instructions below to enter a competing interest statement for this submission. On behalf of all authors, disclose any competing interests that could be perceived to bias this work—acknowledging all financial support and any other relevant financial or non-financial competing interests.<br><br>This statement is required for submission and **will appear in the published article** if the submission is accepted. Please make sure it is accurate and that any funding	he authors have declared that no competing interests exist.

sources listed in your Funding Information later in the submission form are also declared in your Financial Disclosure statement.<br><br>View published research articles from *PLOS ONE* for specific examples.<br><br>**NO authors have competing interests**<br><br>Enter: *The authors have declared that no competing interests exist.*<br><br>**Authors with competing interests**<br><br>Enter competing interest details beginning with this statement:<br><br>*I have read the journal's policy and the authors of this manuscript have the following competing interests: [insert competing interests here]*<br><br>* typeset	
**Ethics Statement**<br><br>Enter an ethics statement for this submission. This statement is required if the study involved:<br><br>• Human participants<br>• Human specimens or tissue<br>• Vertebrate animals or cephalopods<br>• Vertebrate embryos or tissues<br>• Field research<br><br>Write "N/A" if the submission does not require an ethics statement.<br><br>General guidance is provided below. Consult the submission guidelines for detailed instructions. **Make sure that all information entered here is included in the Methods section of the manuscript.**	Approval for this study was obtained from the Institutional Review Board of Madonna University, Nigeria. The study adhered to the tenets of the Declaration of Helsinki and permission to access the patient records was obtained from the management of the Federal Medical Centre (FMC) Gusau, Zamfara State.

**Format for specific study types**<br><br>**Human Subject Research (involving human participants and/or tissue)**<br>• Give the name of the institutional review board or ethics committee that approved the study<br>• Include the approval number and/or a statement indicating approval of this research<br>• Indicate the form of consent obtained (written/oral) or the reason that consent was not obtained (e.g. the data were analyzed anonymously)<br><br>**Animal Research (involving vertebrate animals, embryos or tissues)**<br>• Provide the name of the Institutional Animal Care and Use Committee (IACUC) or other relevant ethics board that reviewed the study protocol, and indicate whether they approved this research or granted a formal waiver of ethical approval<br>• Include an approval number if one was obtained<br>• If the study involved *non-human primates*, add *additional details* about animal welfare and steps taken to ameliorate suffering<br>• If anesthesia, euthanasia, or any kind of animal sacrifice is part of the study, include briefly which substances and/or methods were applied<br><br>**Field Research**<br><br>Include the following details if this study involves the collection of plant, animal, or other materials from a natural setting:<br>• Field permit number<br>• Name of the institution or relevant body that granted permission	
**Data Availability**<br><br>Authors are required to make all data underlying the findings described fully available, without restriction, and from the time of publication. PLOS allows rare exceptions to address legal and ethical concerns. See the PLOS Data Policy and FAQ for detailed information.	Yes - all data are fully available without restriction

A Data Availability Statement describing where the data can be found is required at submission. Your answers to this question constitute the Data Availability Statement and **will be published in the article**, if accepted.<br><br>**Important:** Stating 'data available on request from the author' is not sufficient. If your data are only available upon request, select 'No' for the first question and explain your exceptional situation in the text box.<br><br>Do the authors confirm that all data underlying the findings described in their manuscript are fully available without restriction?	
**Describe where the data may be found in full sentences. If you are copying our sample text, replace any instances of XXX with the appropriate details.**<br><br>• If the data are **held or will be held in a public repository**, include URLs, accession numbers or DOIs. If this information will only be available after acceptance, indicate this by ticking the box below. For example: *All XXX files are available from the XXX database (accession number(s) XXX, XXX.).*<br>• If the data are all contained **within the manuscript and/or Supporting Information files**, enter the following: *All relevant data are within the manuscript and its Supporting Information files.*<br>• If neither of these applies but you are able to provide **details of access elsewhere**, with or without limitations, please do so. For example:<br><br>*Data cannot be shared publicly because of [XXX]. Data are available from the XXX Institutional Data Access / Ethics Committee (contact via XXX) for researchers who meet the criteria for access to confidential data.*<br><br>*The data underlying the results presented in the study are available from (include the name of the third party*	All relevant data are within the manuscript and additional information can be obtained on reasonable request by the Corresponding author

and contact information or URL).<br>• This text is appropriate if the data are owned by a third party and authors do not have permission to share the data.<br><br>* typeset	
Additional data availability information:	

Manuscript Click here to access/download;Manuscript;Main Manuscript.docx

Original article

Prevalence and clinical profile of glaucoma patients in rural Nigeria – A hospital based study

Ngozika E Ezinne[1,2p], Chukwuebuka S Ojukwu[2, 3p], Kingsley K Ekemiri[1†], Obinna F Akano[4†], Edgar Ekure[5†], Uchechukwu L Osuagwu[6,7*p]

Affiliations

[1]Department of Clinical Surgical Sciences, Optometry Unit, University of the West Indies, St Augustine Campus, Trinidad and Tobago,

[2]Department of Optometry, Madonna University, Elele campus, Nigeria

[3]Department of Public Health, Oxford Brookes University, Oxford, United Kingdom

[4]I and Eye Optometry, Bronx, New York, USA

[5]Salus University Pennsylvania, USA

[6*]Diabetes, Obesity and Metabolism Translational Research Unit, School of Medicine, Western Sydney University, Campbelltown, NSW 2560, Australia.

[7]African Vision Research Institute, University of KwaZulu-Natal Durban, Durban, South Africa.

*Corresponding author: Uchechukwu. L. Osuagwu; email: l.osuagwu@westernsydney.edu.au; Tel.: +61-4011-93-234, https://orcid.org/0000-0002-1727-6914

[P] These authors contributed equally to this work

[†] These authors also contributed equally to this work

Acknowledgments: The authors acknowledge the guidance of Late Prof Alabi, O Oduntan during data collection

Abstract

Purpose: To determine the prevalence and clinical presentation of participants with glaucoma attending a public eye care facility in Nigeria

Method: Hospital based retrospective study of glaucoma participants aged 50 years and above seen over a 5-year period. Descriptive statistics summarized the demographic, clinical characteristics and treatment of the participants and determined the association of variables with gender and age. Prevalence of the glaucoma by type, and their 95% confidence intervals (CI) were also calculated.

Result: Of the 5482 case files that were reviewed, 995 (18.15%, 95% Cl 17.15 - 19.19%) had glaucoma particularly primary open angle glaucoma (11.55%, 95%CI 10.73 – 12.42%) and were mostly females (564, 56.7%) aged 69 ± 12 years (range, 50 - 103 years). In contrast to other glaucoma types, the prevalence of primary angle closure glaucoma (3.68, 95%CI 3.22-4.22) increased by 15% over 5 years. The mean intraocular pressure ranged from 15 – 50 mmHg but higher in females than males (27.8 ± 6.1mmHg versus 26.6 ± 6.0 mmHg, $p<0.05$) who had comparable VA (0.58 ± 0.4 Log MAR) and cup-disc ratios ($p>0.05$). On presentation, the glaucoma hemi field test (GHFT) was outside the normal limits in 45.5% and 54.5% of males and females, respectively. The type of visual field defect was associated with glaucoma type (P = 0.047). Arcuate scotoma was most common (35.5%) across glaucoma types, paracentral scotoma more common in Secondary glaucoma while Seidel scotoma was highest in NTG (19.3%). Beta-blocker was the mainstay of management (42.2%) but more likely to be prescribed to males while more females received carbonic anhydrase inhibitors.

Conclusions: The high prevalence of glaucoma in older people remains a public health problem in Nigeria. The fact that about half of the participants presented with visual field defect suggests there

is a need for public health messages to emphasize on early glaucoma screening, detection and management.

Introduction

Glaucoma is a group of disorders characterized by a progressive optic neuropathy resulting in characteristic appearance of the optic disc, irreversible visual field defect that are associated either with elevated intraocular pressure or normal pressure [1]. It is a public health problem accounting for 8% of world blindness and the second leading cause of blindness after cataract [2]. Globally, an estimate of 60.5 million people have glaucoma and about 8.4 million had become blind from the condition [2].

The number of people (aged 40–80 years) with glaucoma has been projected to increase to 111.8 million by 2040 [3-4]. Blindness due to glaucoma can be avoided if the glaucoma is detected early and managed appropriately [5]. The prevalence of glaucoma worldwide is about 1% in older people (aged >50 years) and increases with age [3, 6]. A review of relevant population based surveys of glaucoma, visual impairment and blindness in Sub- Saharan Africa indicate that glaucoma affects about 4% adults aged 40 years and above and accounts for 15% of blindness [5]. The prevalence ranges from 0.66% to 1.79% in Eritrea, Liberia, Ghana, South Africa and Malawi [7-9]. Primary open angle glaucoma (POAG) is the most common form of glaucoma among Africans [5] and contributes to 8.4 million cases of bilateral blindness even in developed countries with half of the cases still undiagnosed [10]. In Nigeria, 1,130,000 individuals' ≥40 years are blind and 4.25 million have moderate to severe visual impairment [11].

Various studies [12-14] in different parts of Nigeria showed that glaucoma is one of the leading causes of blindness in Nigeria with prevalence slightly higher in the Southeastern part of the country. A study in Kano in the northwestern part of Nigeria reported that 15% of blindness and 7% of those visually impaired were due to glaucoma [15]. Also, Murdoch et al [6] reported a prevalence of 1.02% on those above 45 years of age among Hausa/Fulani ethnic group in Nigeria.

There are insufficient glaucoma studies in Nigeria to represent each region and various ethnic group in the country. It is difficult to extrapolate the findings due to differences in cultural and socio-economic activities. There is a need to understand the demographic and clinical presentation of glaucoma in different regions in Nigeria for effective management. Evaluating the epidemiological and clinical profile of glaucoma patients seen at the Federal Medical Centre Eye clinic Gusau, Zamfara State will shed light on inter-ethnic and regional variations of glaucoma prevalence in Nigeria. It will also provide a useful background information for planning epidemiological surveys on glaucoma in this region as well as other parts of Nigeria with similar socio-demographic and ecological characteristics. Therefore, this study was aimed to assess the epidemiological characteristics and clinical presentations of glaucoma patients’ ≥50 years seen at a referral center in Nigeria.

Materials and Methods

Study setting

This retrospective study of adult participants who attended the glaucoma referral center of the Federal Medical Centre (FMC) Gusau, Zamfara State, Nigeria between, January 2011 and December 2016 (5-year period). The eye clinic is one of the two public/government established

eye clinics that serves as a primary health care center for over 3 million residents of Zamfara State and its environs. The region is made up of largely Muslims of Hausa ethnic group many of who (60%) are subsistence farmers that live in rural areas and live in rural areas on less than a dollar per day [17]. There is low literacy level in the region [5, 17-18]. Life expectancy in this region is less than 50 years, there is high poverty rate and the region has ill-equipped hospitals and infrastructure in terms of roads, public transport and access to health care services are relatively poor [18].

Study design and sampling

This was a hospital-based study of participants diagnosed with glaucoma over 5 years. A non-probability convenience sampling method was utilized because all patients with glaucoma who visited the center during the study period were eligible.

Inclusion and exclusion criteria

Data for all participants aged 50 years and over who presented for the first time to this referral center and were diagnosed with glaucoma at the eye clinic during the study period were included. This includes those who had undergone filtration surgery. Exclusion criteria included participants with ocular hypertension, who did not have changes in optic nerve head or visual function abnormalities; those with a history of ocular diseases that could affect the validity of the ocular fundus examination including macular degeneration, retinitis pigmentosa, hypertensive retinopathy, diabetic retinopathy, refractive error of ± 4 Diopter (D) sphere, and/or astigmatism of 3D, and participants with significant cataract that affect vision. Glaucoma participants with incomplete records of C/D ratio, visual field assessment were also excluded from the study.

Techniques for determination of clinical indices of glaucoma

Data collection involved the use of a data extraction sheet to extract information on demographics, and clinical profile of the patients. The data on demographics of patients included gender, age at presentation, ethnic group, religion, and occupation. The clinical profile recorded included presenting visual acuity, IOP, vertical cup-to-disc ratios (VCDR), type of glaucoma, glaucoma hemi field test, type of visual field defect and method of management. Visual acuity was measured using a Snellen chart and converted to logMAR notation for the purpose of analysis. Glaucoma hemi field test (GHFT) was performed with automated Humphrey visual field analyzer (Humphrey 740; Carl Zeiss Meditech, Dublin, CA) but global indices including pattern deviation, mean deviation, pattern standard deviation were not documented at the time; hence, the global indices were not available. IOP was measured using the Goldmann applanation tonometer mounted on a slit lamp bimicroscope and as a routine practice, were taken between the hours of 8 am to 4 pm when the IOP are most stable [19].

For diagnosis of glaucoma, gonioscopy using a Goldmann 3-mirror and fundus eye exam with the Welch-Allyn (Welch-Allyn Inc., Skaneateles Falls, New York, USA) ophthalmoscope was conducted. The hospital used International Society for Geographical and Epidemiological Ophthalmology (ISGEO) for the diagnosis and classification of glaucoma. Glaucomatous optic disc atrophy was confirmed by stereoscopic examination of the optic disc with a +90D lens on the slit lamp. A measuring eyepiece graticle (Haag Streit) was used in measuring the vertical optic diameter and cup diameter. Also noted were the presence of notching on the disc rim and any violation of the ISNT rule. The vertical cup-to-disc ratio (VCDR) was used as an index of structural glaucomatous damage. There was no ocular coherence tomography (OCT) in the hospital at the time of data collection, hence Retinal nerve fiber layer (RNFL) loss and central corneal thickness (CCT) were not included in the study.

Glaucoma diagnosis criteria

The criteria for the classification of glaucoma at this hospital are described below: Criterion 1 Diagnosis (Structural and Functional Evidence) included eyes with a VCDR of 0.7 or more and less than 0.9 and/or VCDR asymmetry of 0.2 or more or a neuroretinal rim width reduced to less than or equal to 0.1 CDR (between 11 and 1 o'clock or 5 and 7 o'clock) that also showed a definite visual field defect consistent with glaucoma. Criterion 2 Diagnosis (Advanced Structural Damage With Unproved Field Loss) included participants who could not satisfactorily complete the visual field test but had eyes with VCDR of 0.9 or more and/or VCDR asymmetry of 0.3 or more. Criterion 3 Diagnosis (Optic Disc Not Seen, Field Test Impossible) was given if it was not possible to examine the optic disc, and eyes had visual acuity less than 20/400, presence of relative afferent pupillary defect with IOP of 26 mm Hg or higher, and/or evidence of glaucoma surgery or medical records confirming glaucomatous visual morbidity [20].

Glaucoma types

Primary Open Angle Glaucoma (POAG) was defined as open and normal appearing angle with IOP $\geq$21 mmHg associated with either glaucomatous optic disc abnormalities (cupping) or glaucomatous visual field abnormalities or with both. Normal tension glaucoma (NTG) was defined as open and normal appearing angle with IOP $\leq$ 21 mmHg at all times, with glaucomatous optic neuropathy or IOP $\leq$ 21 mmHg at all IOP measurements on record. Primary angle closure glaucoma (PACG) was defined as an eye with an occludable drainage angle and features indicating trabecular obstruction by the peripheral iris, such as peripheral anterior synechiae, irido-corneal contact, elevated intraocular pressure (IOP of 21 mmHg or more), together with evidence of glaucomatous optic nerve damage and visual field (VF) loss.

Secondary glaucoma (SG) was defined as raised IOP with glaucomatous optic neuropathy or IOP ≥21mmHg associated with positive history and ocular findings of pathologies such as trauma, previous surgery, neovascularization, inflammation, or any other abnormal ocular or systemic findings that could have caused prior or current IOP elevation**.** In addition, glaucoma, patients with a history of use of topical steroids (6 months), a history of trauma or ocular surgery, chronic uveitis, evidence of pseudo exfoliation or pigment dispersion on slit lamp examination, and those with hyper mature or intumescent cataract were grouped under secondary glaucoma.

Variables Description

The type of Glaucoma (POAG, NTG, PACG and SG) [21] and the clinical indices of glaucoma were the dependent variables at each time. The independent variables included epidemiological characteristics of age, gender, occupation, ethnic groups and religion and clinical indices including type of VF defect, vertical cup-disc ratio (VCDR), IOP, GHFT, VA in LogMar and treatment of the glaucoma (surgery, medications and combinations). For purposes of analysis, participants with counting finger at 2 feet were considered to have a visual acuity of 2/200 or 20/2000. Those with hand movement at a distance of 2 feet were considered to have an equivalent Snellen acuity of 20/20,000. Light perception (LP) with or without projection and no light perception (NLP) are not VA measurements but merely the ability to detect a stimulus. Therefore, these factors were excluded from the analysis.

Ethics

Approval for this study was obtained from the Institutional Review Board of Madonna University, Nigeria. The study adhered to the tenets of the Declaration of Helsinki and

permission to access the patient records was obtained from the management of the Federal Medical Centre (FMC) Gusau, Zamfara State.

Statistical analysis

All data analysis were performed using the IBM SPSS Statistics for Windows, Version 25.0 (IBM Corp., Armonk, NY, USA). Normality distribution of the data was assessed using Kolmogorov–Smirnov test. Data was presented using descriptive statistics using frequencies for categorical variables and mean (±standard deviation, SD; range) for continuous variables. One-way analysis of variance (ANOVA) and chi-square test were performed to assess the differences between groups for the continuous and categorical variables respectively. The differences in the proportion diagnosed with different types of glaucoma by year of diagnosis was also assessed using chi-square test. Univariate analysis was conducted to assess the effects of gender on the clinical indices. The level of statistical significance was set at 5%.

Results

Demographic characteristics of the participants with glaucoma

Of the 5482 casefiles of participants aged 50 years and over who attended this hospital over 5 years period, 995 participants were diagnosed with glaucoma. Table 1 presents the characteristics of this study population indicating that nearly all were Muslims, females (56.7%) and of Hausa origin. The mean age of the participants was 69 ± 12 years (mean ±SD), and about 61% were farmers. The clinical indices, glaucoma hemi field-test classification, type of visual field defect, glaucoma type and treatment in this study population has been shown in Table 1. The table also

shows the mean values for the clinical profiles such as IOP, cup-to-disc ratios, visual acuities and the others.

Table 1 Descriptive statistics of measured variables among glaucoma participants

Variables	n (%)
**Demography** n(%)	995/5482 (18.2%)
Age, mean (SD)	69.2 (11.8), 50-103
Gender	
Male	431 (43.3)
Female	564 (56.7)
Ethnic group	
Fulani	183 (18.4)
Hausa	631 (63.4)
Others	178 (17.9)
African Traditional	9 (1.0)
Christian	84 (8.4)
Islam	901 (90.6)
Occupation	
Employed	90 (9.0)
Farming	613 (61.6)
Retired	181 (18.2)
Self employed	111 (11.2)
**Clinical index,** mean (SD), range	
Visual acuity (RE)	0.58 (0.40), 0-2.8
Visual acuity (LE)	0.55 (0.38), 0-2.8
Cup-disc ratio (RE)	0.69 (0.11), 0.30-0.90
Cup-disc ratio (LE)	0.69 (0.12), 0.3-0.9
Intraocular pressure (RE)	27 (6), 15-45
Intraocular pressure (LE)	27 (6), 15-50
**Glaucoma Hemi field Test**	
Borderline	231 (23.2)
Outside Normal Limit	541 (54.4)
Reduced Sensitivity	55 (5.5)
Within Normal Limits	168 (16.9)
**Visual field Defects**	
Arcuate	353 (35.5)
Paracentral	52 (5.2)
Ring	224 (22.5)
Seidel	98 (9.8)
Tunnel	268 (26.9)

**Glaucoma type**	
Normal tension	57 (5.7)
Primary angle closure	202 (20.3)
Primary open angle	633 (63.6)
Secondary	103 (10.4)
**Treatment**	
Surgery only	18 (1.8)
Trabeculectomy + Alpha 2 agonist	49 (4.9)
Trabeculectomy + prostaglandin analogues	10 (1)
Trabeculectomy + Beta-blocker	43 (4.3)
Prostaglandin analogue	112 (11.3)
Carbonic anhydrase inhibitor	78 (7.8)
Beta blocker	420 (42.2)
Alpha 2 agonist	265 (26.6)

VA was recorded in Log MAR= logarithmic minimum angle of resolution; SD= standard deviation; RE=right eye; LE=left eye.

Of clinical indices, VA was drastically reduced with mean VA of 0.58 ± 0.4 LogMAR indicating visual impairment. There were 23 (2.31%) and 6 participants (0.60%) whose VA in either or both eyes respectively was recorded as counting finger (n=1, 4.3%), hand movement (n=9, 0.90%), and light perception (15, 1.5%). For 375 participants (37.7%), VA in the better Seeing Eye was worse than 0.5LogMAR indicating either low vision (n=315, 31.6%) or blindness (n=60, 6.0%) according to the WHO definition for blindness as a best-corrected visual acuity worse than 1.3 LogMAR.

The mean IOP in this study group ranged from 15 - 50mmHg with an average cup-disc ratio of 0.7. For majority of the participants, beta-blocker was the mainstay of therapy (42.2%) and about 1.8% had glaucoma filtration surgery done. Arcuate and ring scotomas were the predominant visual field defect among the participants consisting of about 58% of the reported visual field defects.

Prevalence of Glaucoma

Figure 1 shows the prevalence by glaucoma type over 5 years in this rural referral hospital. Over the five-year study period, 18.15% [95% Confidence interval CI 17.15 - 19.19] had glaucoma in this referral hospital. The highest prevalence was for POAG, which was more than three times higher than that of PACG. The lowest prevalence was for NTG.

Figure 1: **Prevalence of Glaucoma by type over 5 years.** ***Error bars represent 95% confidence intervals***.

Analysis of glaucoma type

Chi-square analysis revealed no significant association between the type of glaucoma and the demographic factors of gender (p=0.122), occupation (p=0.169), and ethnic group (p=0.408), but age and year of glaucoma diagnosis were associated with glaucoma type in this study group (p<0.0005, for both). Participants who were diagnosed with NTG were younger (57 ± 9 years) than those in PACG (71 ± 11 years), PAOG (70 ± 12 years), and SG (69 ± 12 years) groups (p<0.0005, for all comparisons).

Figure 2 presents the glaucoma types by year of diagnosis showing that except for PACG, which increased by about 15% over the five-year period, all other glaucoma types showed a decline in the proportion diagnosed over 5 years. Overall, 50% fewer cases were diagnosed with glaucoma in 2016 compared with 2011, in this rural hospital.

Figure 2: Percentage distribution of glaucoma type by year of diagnosis

The type of visual field defect was also associated with glaucoma type (*P* = 0.047) as shown in Figure 3, with arcuate scotoma (35.5%) being the most predominant visual field defect across all types of glaucoma, followed by tunnel vision. Although fewer people had paracentral scotoma, it was more among those diagnosed with SG and POAG. Seidel scotoma was highest among those diagnosed with NTG (19.3%).

Figure 3. Percentage distribution of the visual field defect by glaucoma type

Analysis of the clinical profiles and treatment types

The mean values for the clinical profiles by gender is shown in Table 2. The mean IOP (27± 6 mmHg) was significantly higher in females than males (27.8 ± 6.1mmHg versus 26.6 ± 6.0 mmHg, p<0.05) who had comparable VA and cup-disc ratios (p>0.05). For more than half of the participants (n=541, 54.4%), the glaucoma hemi field test was outside the normal limit and it was within normal limits for 16.9% of the participants (Table 1) and comparable between gender (Table 2, *P*=0.136).

Table 2: Clinical indices and treatment of glaucoma participants aged 50 years and over

Variables	Male	Female	P-Value
**Clinical index, mean (SD), range**	RE/LE	RE/LE	
Visual acuity (RE)	0.58 (0.42)/0.56 (0.40)	0.57 (0.39)/0.55 (0.35)	0.799, 0.661
Cup-disc ratio	0.68 (0.11)/0.68 (0.11)	0.69 (0.10)/0.69 (0.12)	0.268, 0.322
Intraocular pressure (RE)	26.6 (6.0)/26.3 (5.9)	27.8 (6.10)/27.4 (5.97)	0.002, 0.006
**Glaucoma Hemi field Test, n (%)**			
Borderline	103 (44.6)	128 (55.4)	0.136
Outside Normal Limit	246 (45.5)	295 (54.5)	
Reduced Sensitivity	18 (32.7)	37 (67.3)	
Within Normal Limits	64 (38.1)	104 (61.9)	

**Treatment, n (%)**			
Trabeculectomy only	10 (2.3)	8 (1.4)	0.021
Trabeculectomy + Alpha 2 agonist	27 (6.3)	22 (3.9)	
Trabeculectomy + prostaglandin analogues	4 (0.9)	6 (1.1)	
Trabeculectomy + Beta-blocker	24 (5.6)	19 (3.4)	
Prostaglandin analogue	51 (11.8)	61 (10.8)	
Carbonic anhydrase inhibitor	44 (10.2)	34 (6.0)	
Beta blocker	169 (39.2)	251 (44.5)	
Alpha 2 agonist	102 (23.7)	163 (28.9)	

VA was recorded in Log MAR= logarithmic minimum angle of resolution; SD= standard deviation; RE=right eye and LE=left eye were for clinical index only..

The treatment type varied significantly between males and females. Males were more likely to be treated with Alpha 2 agonist and beta-blockers, while females were more likely to receive carbonic anhydrase inhibitors (Table 2). About 12.1% of participants had done glaucoma filtration surgery (Trabeculectomy) for control of intraocular pressure and more in males than females (n=55, 15.1% versus n=47, 9.8%).

Discussion

In the present study, epidemiological and clinical profile of glaucoma patients 50 years and above seen at a health care facility for a period of 5 years were evaluated. There was a high prevalence of glaucoma particularly open angle glaucoma, especially among females, Muslims and farmers. Whereas there was a decline in prevalence for other types of glaucoma, the prevalence of PACG in this underserved community increased by 15% over 5 years. Contrary to a previous report [22], the prevalence of PACG exceeded that of NTG by about 4 folds. The type of visual field defect varied significantly with the glaucoma type but arcuate scotoma was most common in all glaucoma types. Although, beta-blocker was the main drug of choice of glaucoma treatment in

this hospital, men were more likely to receive this treatment than women who were more likely to receive carbonic anhydrase inhibitors. At the time of this study, about a quarter of the participants, more men than women (15% versus 10%) already had Trabeculectomy as a surgical procedure for control of their intraocular pressures.

The prevalence of glaucoma reported in this region was considerably higher than previous estimates from survey studies (ranging from 1% to 8.6%) in other parts of the country [14, 20, 23-25]. Such high prevalence is expected since this region has only two primary health care centers that provide eye care services; therefore high influx of patients will be expected at this center. The fact that our study was in the northern part of Nigeria where majority of the participants were of Hausa ethnic group (less educated) may contribute to the difference in prevalence compared with other studies which included the more educated ethnic groups (Yorubas and Igbos) [20, 24]. Also, the lack of awareness and poor utilization of eye care services reported in some parts of Nigeria [26-28] could be the reason for the reduced prevalence recorded. There is a need for more awareness to be created and more eye care outlet established in underserved communities in Nigeria to encourage utilization of eye care services.

Similar to the present report, high prevalence of POAG has been reported in the black race including among African Americans and Afro-Caribbean [5] and in other studies [4, 29-34]. It is possible that the prevalence reported in our study may have been underestimated as POAG is usually asymptomatic and people only seek for medical attention when it becomes severe and affect vision. Although the prevalence of POAG observed in this study was higher than previous reports from Nigeria [5, 14, 18, 24, 25, 35], it was much lower than the 91.2% recorded in another hospital based study from Benin City [36]. Considering the rurality of this community, there is a high possibility that many remain cases of PAOG remain undetected in this population.

The present finding of a significant increase in PACG prevalence during the study period is in agreement with the projected global increase in the prevalence of PACG (from 23 - 32 million over the next 2 decades [37]. Also, the prevalence of PACG in this Northern hospital exceeds the 1.7% that was reported in Southern hospital studies [14, 25]. The study found a marked reduction in the prevalence of all other glaucoma types including POAG, which might not necessarily reflect reduction in glaucoma prevalence but rather a decrease in the utilization of eye care services triggered by insurgency and civil unrest predominant in this region [18]. In addition to these factors, poor awareness of glaucoma and low life expectancy in Nigeria could play a role in the decline in glaucoma prevalence [18, 38]. Contrary to our findings, a hospital-based study in Benin City recorded a monthly increase in glaucoma prevalence from 10 to 27% [36] but failed to distinguish between glaucoma types. This increase might be attributed to greater glaucoma awareness, and higher socioeconomic status of the participants since data was from a private owned hospital. However, in another study conducted in a South Korean public hospital, a 54% annual increase in glaucoma prevalence was observed over 5 years. This increase could be attributed to the improvement in glaucoma detection techniques at this hospital, as well as increase in access to eye care services (increased by 9%) and the life span of people in the region (increased by 14.28%) [34].

There are mixed reports on the effect of gender on glaucoma prevalence. The present study found no significant difference in glaucoma prevalence between male and females, which was similar to previous studies from Ghana [39-40]. In contrast, studies from Nigeria [5, 35-36], Ghana [41] and South Korea [39] reported a higher prevalence in men than women. Moreover, gender predilection of glaucoma has not been established suggesting the need for more studies to determine the association of glaucoma with gender. Age is a risk factor for glaucoma [42-45] and

this was also associated with glaucoma type in this study. Participants with NTG were younger than other glaucoma types even though the overall mean age of participants in this study was similar to previous studies [14, 25, 36, 46-48]. This finding further confirms the importance of visual field and optic nerve assessment as part of the early screening of glaucoma in this population.

The mean VCDR recorded in this study was similar to that of the national eye survey in Nigeria [49], but less than the VCDR recorded among participants in Oyo State Nigeria [20], Tanzania [50] and Netherland [51]. There is a limited information on the distribution of VCDR among Nigerian population; although those from Igbo ethnic group have larger optic disc area and cup than other ethnic groups [5]. The visual field defect, which is one of the hallmark used in the diagnosis of glaucoma, occurs as a result of optic disc cupping. For a good number of the participants in the present study, the glaucoma hemi field test was outside the normal limit field. Uncontrolled IOP due to late presentation could be the reason for the increased visual field loss recorded in this study [52]. Furthermore, the rate of progression of the visual field defect varies in patients, and treatment of the glaucoma may not completely stop the visual field loss as some patients still progress despite treatment. Early screening for glaucoma is highly indicated in this region. Majority of the participants in this study presented to the clinic at the late stage of glaucoma with many already having significant visual field loss leading to tunnel vision or blindness in at least one eye, which confirms the findings of other studies in Africa [18].

That a good number of participants in this study had severe visual impairment and blindness on first presentation to the clinic was in line with previous reports from Nigeria [5, 28, 35, 11, 53] and Saudi Arabia [54]. In North-eastern Nigeria, a study found that about 76% were already blind at hospital presentation. Old age, poor knowledge of glaucoma, rural residence and

living far from the hospital were attributed to the late presentation of glaucoma patients in Nigeria [18, 53]. In addition, the report of earlier age of onset of glaucoma among Africans or black population may contribute to the high rate of blindness in this population since they would have had the disease for a longer time [35]. Public eye health education and glaucoma screening programs in the rural communities in Nigeria cannot be over emphasized. The Nigerian government should consider ameliorating programs aimed at reducing cost for glaucoma management especially in this region.

The uptake of glaucoma surgery in this region was low and could be attributed to the reported low success rate of Trabeculectomy among black patients [6]. Inadequate access, high cost of surgery, superstition and socio-cultural beliefs may contribute to the preference for medical treatment rather than surgery [55]. In Ethiopia, authors reported a high uptake of glaucoma surgery [56] as ophthalmologists in the country choose surgery over medications due to patients' non-compliance. Similar to a study in Ghana [46], we found that beta-blockers such as timolol were the mainstay of treatment. This could be explained by the fact that it is more affordable and readily available than other classes of drugs including prostaglandin analogues (latanoprost), which are considered the first line of treatment for lowering IOP [57]. In addition, prostaglandin analogues have ocular adverse effects like pruritus, conjunctival hyperemia, ocular irritation, ocular pain, burning, and cilia alteration which may not be pleasant in older people.

Strengths and Limitation

The study has some limitations. First, as a single hospital-based study, the findings are better representatives of the clinical situation compared with population studies but the findings cannot be representative of the general population in Northern Nigeria or the country at large. A population based study is needed with a larger number of patients, to substantiate information

obtained from this study. Also, we did not assess associations with other ocular conditions like myopia and comorbid conditions which would require further investigation with additional hospital based data. Retinal nerve fibre layer loss and central corneal thickness were skipped in the diagnosis due to the unavailability of OCT data at the hospital during the period of data collection. The fact that OCT was not used in the glaucoma diagnosis could have affected the low prevalence of NTG. Normal tension glaucoma (NTG) may be very difficult to detect without OCT and/or pachymetry because it occurs with normal IOP. Despite the limitations, our study is the first to highlight the epidemiology of glaucoma in this region and the key findings were comparable with results from other studies.

Conclusion

This study found that among people aged 50 years and above in this underserved community, the prevalence of glaucoma was higher than previously reported in other parts of Nigeria. Although primary open angle glaucoma (POAG) showed a decline, it remains a public health problem in Nigeria together with the added burden from the increasing rate of angle closure glaucoma in this community. The fact that majority of the participants with glaucoma in this region still present late when their ganglion cells and vision have already been severely affected calls for urgent public health measures for glaucoma control in this region. Public health messages emphasizing on early glaucoma screening, detection and management are needed.

Declarations

Funding: This research did not receive any funding.

Conflicts of interest/Competing interests: The authors have no financial discloures to make and no conflict of interest

Availability of data and material: The data that support the findings of this study are available on request from the corresponding author, ULO.

Authors' contributions: All authors made substantial contributions to the conception or design of the work; or the acquisition, analysis, or interpretation of data for the work.

References

1. Foster PJ, Buhrmann R, Quigley HA, Johnson GJ. The definition and classification of glaucoma in prevalence surveys. Br J Ophthalmol. 2002; 86(2): 238-242. doi: 10.1136/bjo.86.2.238. PMID: 11815354; PMCID: PMC1771026.
2. Bourne R, Steinmetz JD, Flaxman S, Briant PS, Taylor HR, Resnikoff S, et al. Trends in prevalence of blindness and distance and near vision impairment over 30 years: An analysis for the global burden of disease study. Lancet Glob Health. 2021; 9(2):e144-e160. doi: 10.1016/S2214-109X(20)30425-3. PMID: 33275950; PMCID: PMC7820390.
3. Quigley HA, Broman AT. The number of people with glaucoma worldwide in 2010 and 2020. Br J Ophthalmol. 2006; 90(3):262-7. doi: 10.1136/bjo.2005.081224. PMID: 16488940; PMCID: PMC1856963.
4. Tham YC, Li X, Wong TY, Quigley HA, Aung T, Cheng CY. Global prevalence of glaucoma and projections of glaucoma burden through 2040: a systematic review and meta-analysis. Ophthalmology. 2014; 121(11):2081-90. doi: 10.1016/j.ophtha.2014.05.013. PMID: 24974815.
5. Kyari F, Entekume G, Rabiu M, Spry P, Wormald R, Nolan W, et al. A Population-based survey of the prevalence and types of glaucoma in Nigeria: results from the Nigeria

National Blindness and Visual Impairment Survey. BMC Ophthalmol. 2015; 15: 176. doi: 10.1186/s12886-015-0160-6. PMID: 26653326; PMCID: PMC4676891.

6. Bastawrous A, Burgess PI, Mahdi AM, Kyari F, Burton MJ, Kuper H. Posterior segment eye disease in sub-Saharan Africa: review of recent population-based studies. Trop Med Int Health. 2014; 19(5):600-9. doi: 10.1111/tmi.12276. PMID: 24479434; PMCID: PMC4065367.
7. Kyari F, Abdull MM, Wormald R, Evans JR, Nolan W, Murthy GV, et al. Risk factors for open-angle glaucoma in Nigeria: results from the Nigeria National Blindness and Visual Impairment Survey. BMC Ophthalmol. 2016; 16:78. doi: 10.1186/s12886-016-0264-7. PMID: 27267038; PMCID: PMC4895902.
8. Budenz DL, Barton K, Whiteside-de Vos J, Schiffman J, Bandi J, Nolan W, et al. Prevalence of glaucoma in an urban West African population: the Tema Eye Survey. JAMA Ophthalmol. 2013; 131(5):651-8. doi: 10.1001/jamaophthalmol.2013.1686. PMID: 23538512; PMCID: PMC4139110.
9. Rotchford AP, Kirwan JF, Muller MA, Johnson GJ, Roux P. Temba glaucoma study: a population-based cross-sectional survey in urban South Africa. Ophthalmology. 2003; 110(2):376-82. doi: 10.1016/S0161-6420(02)01568-3. PMID: 12578784.
10. Quigley HA. Glaucoma. Lancet. 2011; 377(9774):1367-77. doi: 10.1016/S0140-6736(10)61423-7. PMID: 21453963.
11. Duke R, Akinye A, Ameh S. Presenting Visual Acuity and Ocular Comorbidity in Patients with Primary Open Angle Glaucoma in a Private Tertiary Eye Center in Nigeria. J Curr Glaucoma Pract. 2013; 7(1):6-10. doi: 10.5005/jp-journals-10008-1129.PMID: 26997773; PMCID: PMC4741127.

12. Enock ME, Omoti AE, Momoh RO. Glaucoma in a suburban tertiary care hospital in Nigeria. J Ophthalmic Vis Res. 2010; 5(2):87-91. PMID: 22737336; PMCID: PMC3380680.

13. Asana UE, Megbelayin EO, Ibanga AA, Nkanga DG, Duke RE, Etim BA. Challenges in the management of glaucoma in university of Calabar teaching hospital, Calabar, Nigeria: A 10 year review. Arch Int Surg 2013; 3: 23-28. DOI: 10.4103/2278-9596.117140

14. Omoti AE, Osahon AI, Waziri-Erameh MJ. Pattern of presentation of primary open-angle glaucoma in Benin City, Nigeria. Trop Doct. 2006; 36(2):97-100. doi: 10.1258/004947506776593323. PMID: 16611443.

15. Abdu L. Prevalence and causes of blindness and low vision in Dambatta local government area, Kano State, Nigeria. Niger J Med. 2002; 11(3):108-12. PMID: 12221951.

16. Murdoch IE, Cousens SN, Babalola OE, Yang YF, Abiose A, Jones BR. Glaucoma prevalence may not be uniformly high in all 'black' populations. Afr J Med Med Sci. 2001; 30(4):337-9. PMID: 14510115.

17. Ibrahim N, Pozo-Martin F, Gilbert C. Direct non-medical costs double the total direct costs to patients undergoing cataract surgery in Zamfara state, Northern Nigeria: a case series. BMC Health Serv Res. 2015; 15:163. doi: 10.1186/s12913-015-0831-2. PMID: 25881013; PMCID: PMC4404068.

18. Statistics | At a glance: Nigeria | UNICEF. Available at http://www.unicef.org/infobycountry/nigeria_statistics.html.

19. Lee YR, Kook MS, Joe SG, Na JH, Han S, Kim S, et al. Circadian (24-hour) pattern of intraocular pressure and visual field damage in eyes with normal-tension glaucoma. Invest Ophthalmol Vis Sci. 2012; 53: 881–887. PMID:22266515

20. Ashaye A, Ashaolu O, Komolafe O, Ajayi BG, Olawoye O, Olusanya B, et al. Prevalence and types of glaucoma among an indigenous African population in southwestern Nigeria. Invest Ophthalmol Vis Sci. 2013; 54(12):7410-6. doi: 10.1167/iovs.13-12698. PMID: 24135752.

21. Lee DA, Higginbotham EJ. Glaucoma and its treatment: a review. Am J Health Syst Pharm. 2005; 62(7):691-9. doi: 10.1093/ajhp/62.7.691. PMID: 15790795.

22. Rotchford AP, Johnson GJ. Glaucoma in Zulus: a population-based cross-sectional survey in a rural district in South Africa. Arch Ophthalmol. 2002; 120(4):471-8. doi: 10.1001/archopht.120.4.471. PMID: 11934321.

23. Abdull MM, Sivasubramaniam S, Murthy GV, Gilbert C, Abubakar T, Ezelum C, et al. Causes of blindness and visual impairment in Nigeria: the Nigeria national blindness and visual impairment survey. Invest Ophthalmol Vis Sci. 2009; 50(9):4114-20. doi: 10.1167/iovs.09-3507. PMID: 19387071.

24. Ekwerekwu CM, Umeh RE. The prevalence of glaucoma in an onchoendemic community in South-Eastern Nigeria. West Afr J Med. 2002; 21(3):200-3. doi: 10.4314/wajm.v21i3.28029. PMID: 12744567.

25. Adio AO, Onua AA. Economic burden of glaucoma in Rivers State, Nigeria. Clin Ophthalmol. 2012; 6:2023-31. doi: 10.2147/OPTH.S37145. PMID: 23271881; PMCID: PMC3526906.

26. Mbadugha CA, Onakoya AO. The awareness, perceptions and experiences of primary open angle glaucoma patients in Lagos Nigeria. Sci Rep. 2014; 4:7585. doi: 10.1038/srep07585. PMID: 25533382; PMCID: PMC4650957.

27. Isawumi MA, Hassan MB, Akinwusi PO, Adebimpe OW, Asekun-Olarinmoye EO, Christopher AC, et al. Awareness of and Attitude towards glaucoma among an adult rural population of Osun State, Southwest Nigeria. Middle East Afr J Ophthalmol. 2014; 21(2):165-9. doi: 10.4103/0974-9233.129769. PMID: 24791109; PMCID: PMC4005182.

28. Kizor-Akaraiwe NN, Monye HI, Okeke S. Awareness and knowledge about glaucoma and proportion of people with glaucoma in an urban outreach programme in Southeast Nigeria. BMJ Open Ophthalmol. 2017; 1(1):e000018. doi: 10.1136/bmjophth-2016-000018. PMID: 29354697; PMCID: PMC5721634.

29. Yoon KC, Choi W, Lee HS, Kim SD, Kim SH, et al. An Overview of Ophthalmologic Survey Methodology in the 2008–2015 Korean National Health and Nutrition Examination Surveys. Korean J Ophthalmol. 2015; 29:359–367. 10.3341/kjo.2015.29.6.359

30. Resnikoff S, Pascolini D, Etya'ale D, Kocur I, Pararajasegaram R, Pokharel GP, et al. Global data on visual impairment in the year 2002. Bull World Health Organ. 2004; 82(11):844-51. PMID: 15640920; PMCID: PMC2623053.

31. Kim MJ, Kim MJ, Kim HS, Jeoung JW, Park KH. Risk factors for open-angle glaucoma with normal baseline intraocular pressure in a young population: the Korea National Health and Nutrition Examination Survey. Clin Exp Ophthalmol. 2014; 42(9):825-32. doi: 10.1111/ceo.12347. PMID: 24735011.

32. Cho HK, Kee C. Population-based glaucoma prevalence studies in Asians. Surv Ophthalmol. 2014; 59(4):434-47. doi: 10.1016/j.survophthal.2013.09.00. PMID: 24837853.

33. Budenz DL, Barton K, Whiteside-de Vos J, et al. Prevalence of Glaucoma in an Urban West African Population: The Tema Eye Survey. JAMA Ophthalmol. 2013; 131(5):651–658. doi:10.1001/jamaophthalmol.2013.1686

34. Seo SJ, Lee YH, Lee SY, Bae HW, Hong S, Seong GJ, et al. Estimated Prevalence of Glaucoma in South Korea Using the National Claims Database. Journal of ophthalmology. 2016; 2016: 1690256. https://doi.org/10.1155/2016/1690256

35. Olawoye O, Tarella S. Spectrum of glaucoma presentation in a Nigerian tertiary hospital. Niger J Ophthalmol 2014; 22: 11-15. DOI: 10.4103/0189-9171.142747

36. Usifoh SF, Udezi AW, Omage OJ. Prevalence of glaucoma in a tertiary hospital. J pharm Biores 2014; 11 (1): 22-28. DOI: 10.4314/jpb.v11i1.4.

37. Zhang N, Wang J, Chen B, Li Y, Jiang B. Prevalence of Primary Angle Closure Glaucoma in the Last 20 Years: A Meta-Analysis and Systematic Review. Front Med (Lausanne). 2021; 7:624179. doi: 10.3389/fmed.2020.624179. PMID: 33537335; PMCID: PMC7847989.

38. Komolafe OO, Omolase CO, Bekibele CO, Ogunleye OA, Komolafe OA, Omotayo FO. Awareness and knowledge of glaucoma among workers in a Nigerian tertiary health care institution. Middle East Afr J Ophthalmol. 2013; 20(2):163-7. doi: 10.4103/0974-9233.110609. PMID: 23741136; PMCID: PMC3669494.

39. Gyasi ME, Francis AW, Chen Y, Harrison RS, Kodjo AR. Presentation of glaucoma in the greater Accra metropolitan area of Ghana. Ghana Med J. 2014; 48(3):143-7. doi: 10.4314/gmj.v48i3.4. PMID: 25709123; PMCID: PMC4335450.
40. Ntim-Amponsah CT, Amoaku WM, Ofosu-Amaah S, Ewusi RK, Idirisuriya-Khair R, Nyatepe-Coo E, et al. Prevalence of glaucoma in an African population. Eye (Lond). 2004; 18(5):491-7. doi: 10.1038/sj.eye.6700674. PMID: 15131680.
41. Kyei S, Obeng PA, Kwarteng MA, Assiamah F. Epidemiology and clinical presentation of glaucoma in a referral facility in Ghana: Any lessons for public health intervention? PLoS One. 2021; 16(1):e0245486. doi: 10.1371/journal.pone.0245486. PMID: 33449975; PMCID: PMC7810334.
42. Hashemi H, Mohammadi M, Zandvakil N, Khabazkhoob M, Emamian MH, Shariati M, et al. Prevalence and risk factors of glaucoma in an adult population from Shahroud, Iran. J Curr Ophthalmol. 2018; 31(4):366-372. doi: 10.1016/j.joco.2018.05.003. PMID: 31844784; PMCID: PMC6896457.
43. Yamamoto S, Sawaguchi S, Iwase A, Yamamoto T, Abe H, Tomita G, et al. Primary open-angle glaucoma in a population associated with high prevalence of primary angle-closure glaucoma: the Kumejima Study. Ophthalmology. 2014; 121(8):1558-65. doi: 10.1016/j.ophtha.2014.03.003.
44. Sun J, Zhou X, Kang Y, Yan L, Sun X, Sui H, et al. Prevalence and risk factors for primary open-angle glaucoma in a rural northeast China population: a population-based survey in Bin County, Harbin. Eye (Lond). 2012; 26(2):283-91. doi: 10.1038/eye.2011.243. PMID: 22157917; PMCID: PMC3272184.

45. Liang Y, Friedman DS, Zhou Q, Yang XH, Sun LP, Guo L, et al. Prevalence and characteristics of primary angle-closure diseases in a rural adult Chinese population: the Handan Eye Study. Invest Ophthalmol Vis Sci. 2011; 52(12):8672-9. doi: 10.1167/iovs.11-7480. PMID: 21908580.

46. Nelson-Ayifah D, Mashige KP. Demographic and clinical characteristics of patients with glaucoma in a tertiary eye facility in Ghana. Afr Vision Eye Health. 2020; 79(1), a521. https://doi.org/10.4102/aveh.v79i1.521

47. Al Obeidan SA, Dewedar A, Osman EA, Mousa A. The profile of glaucoma in a Tertiary Ophthalmic University Center in Riyadh, Saudi Arabia. Saudi J Ophthalmol. 2011; 25(4):373-9. doi: 10.1016/j.sjopt.2011.09.001. PMID: 23960951; PMCID: PMC3729326.

48. Agarwal R, Gupta SK, Agarwal P, Saxena R, Agrawal SS. Current concepts in the pathophysiology of glaucoma. Indian J Ophthalmol. 2009; 57(4):257-66. doi: 10.4103/0301-4738.53049. PMID: 19574692; PMCID: PMC2712693.

49. Kyari F, Tafida A, Sivasubramaniam S, Murthy GV, Peto T, Gilbert CE, et al. Prevalence and risk factors for diabetes and diabetic retinopathy: results from the Nigeria national blindness and visual impairment survey. BMC Public Health. 2014; 14:1299. doi: 10.1186/1471-2458-14-1299. PMID: 25523434; PMCID: PMC4301086.

50. Buhrmann RR, Quigley HA, Barron Y, West SK, Oliva MS, Mmbaga BB. Prevalence of glaucoma in a rural East African population. Invest Ophthalmol Vis Sci. 2000; 41(1):40-8. PMID: 10634599.

51. Wolfs RC, Borger PH, Ramrattan RS, Klaver CC, Hulsman CA, Hofman A, et al. Changing views on open-angle glaucoma: definitions and prevalences--The Rotterdam Study. Invest Ophthalmol Vis Sci. 2000; 41(11):3309-21. PMID: 11006219.

52. Kapetanakis VV, Chan MP, Foster PJ, Cook DG, Owen CG, Rudnicka AR. Global variations and time trends in the prevalence of primary open angle glaucoma (POAG): a systematic review and meta-analysis. Br J Ophthalmol. 2016; 100(1):86-93. doi: 10.1136/bjophthalmol-2015-307223. PMID: 26286821; PMCID: PMC4717368.

53. Kyari F, Abdull MM, Wormald R, Evans JR, Nolan W, Murthy GV, et al. Risk factors for open-angle glaucoma in Nigeria: results from the Nigeria National Blindness and Visual Impairment Survey. BMC Ophthalmol. 2016; 16:78. doi: 10.1186/s12886-016-0264-7. PMID: 27267038; PMCID: PMC4895902.

54. Khandekar R, Chauhan D, Yasir ZH, Al-Zobidi M, Judaibi R, Edward DP. The prevalence and determinants of glaucoma among 40 years and older Saudi residents in the Riyadh Governorate (except the Capital) - A community based survey. Saudi J Ophthalmol. 2019; 33(4):332-337. doi: 10.1016/j.sjopt.2019.02.006. PMID: 31920442; PMCID: PMC6950957.

55. Ocansey S, Kyei S, Diafo A, Darfor KN, Boadi-Kusi SB, Aglobitse PB. Cost of the medical management and prescription pattern for primary open angle glaucoma (POAG) in Ghana- a retrospective cross-sectional study from three referral facilities. BMC Health Serv Res. 2016; 16: 282. PMID:27430262

56. Alemu AM, Nelson LA, Kruft B, Stewart JA, Stewart WC. Epidemiology of glaucoma in central Ethiopia. Int J Ophthalmol. 2009; 2:168–173.

57. Lafuma A, Berdeaux G. Costs and effectiveness of travoprost versus a dorzolamide + timolol fixed combination in first-line treatment of glaucoma: Analysis conducted on the United Kingdom General Practitioner Research Database. Curr Med Res Opin. 2007; 23(12):3009–3016. https://doi.org/10.1185/030079907X242836

Figure 1

Click here to access/download;Figure;Fig 1.tiff

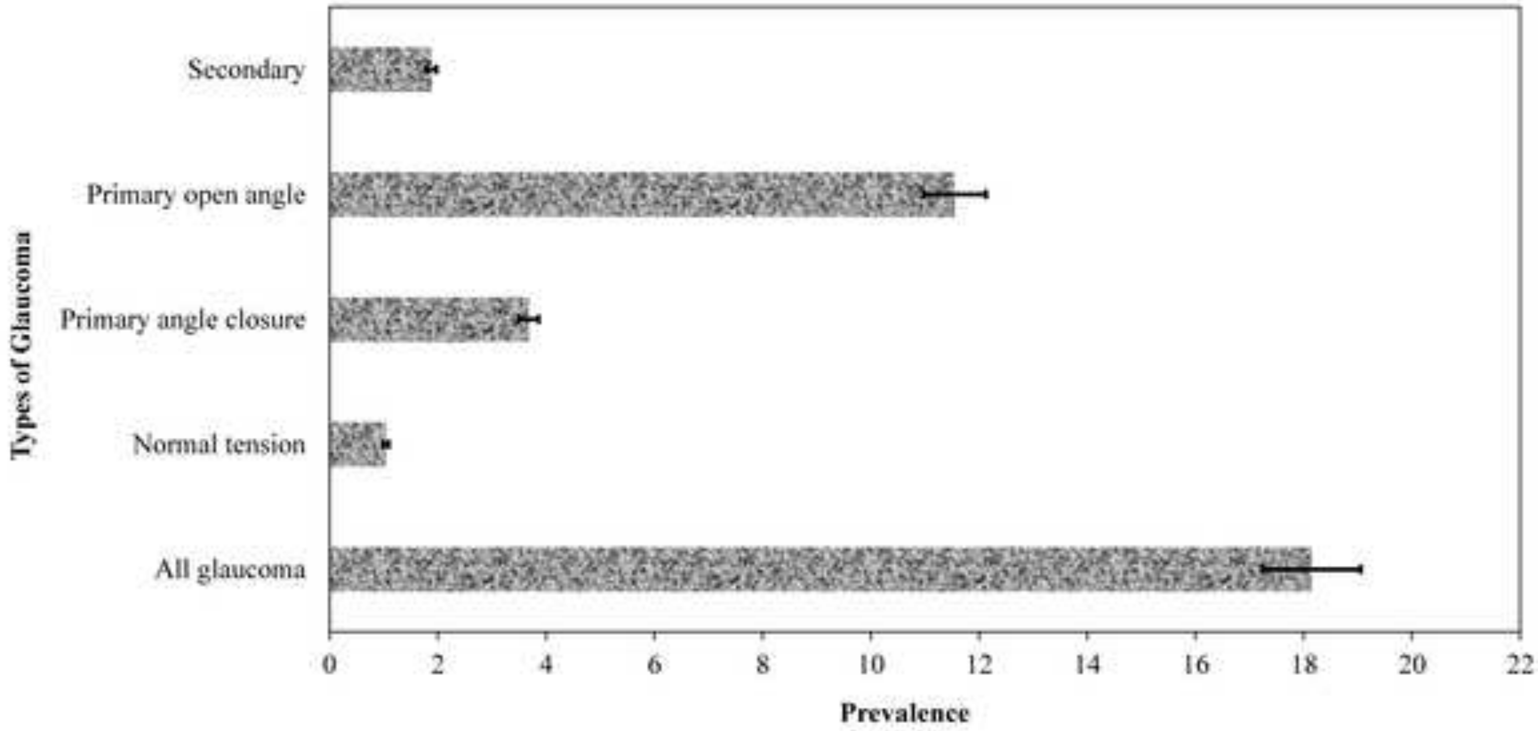

Figure 2

Click here to access/download;Figure;Fig 2.tif

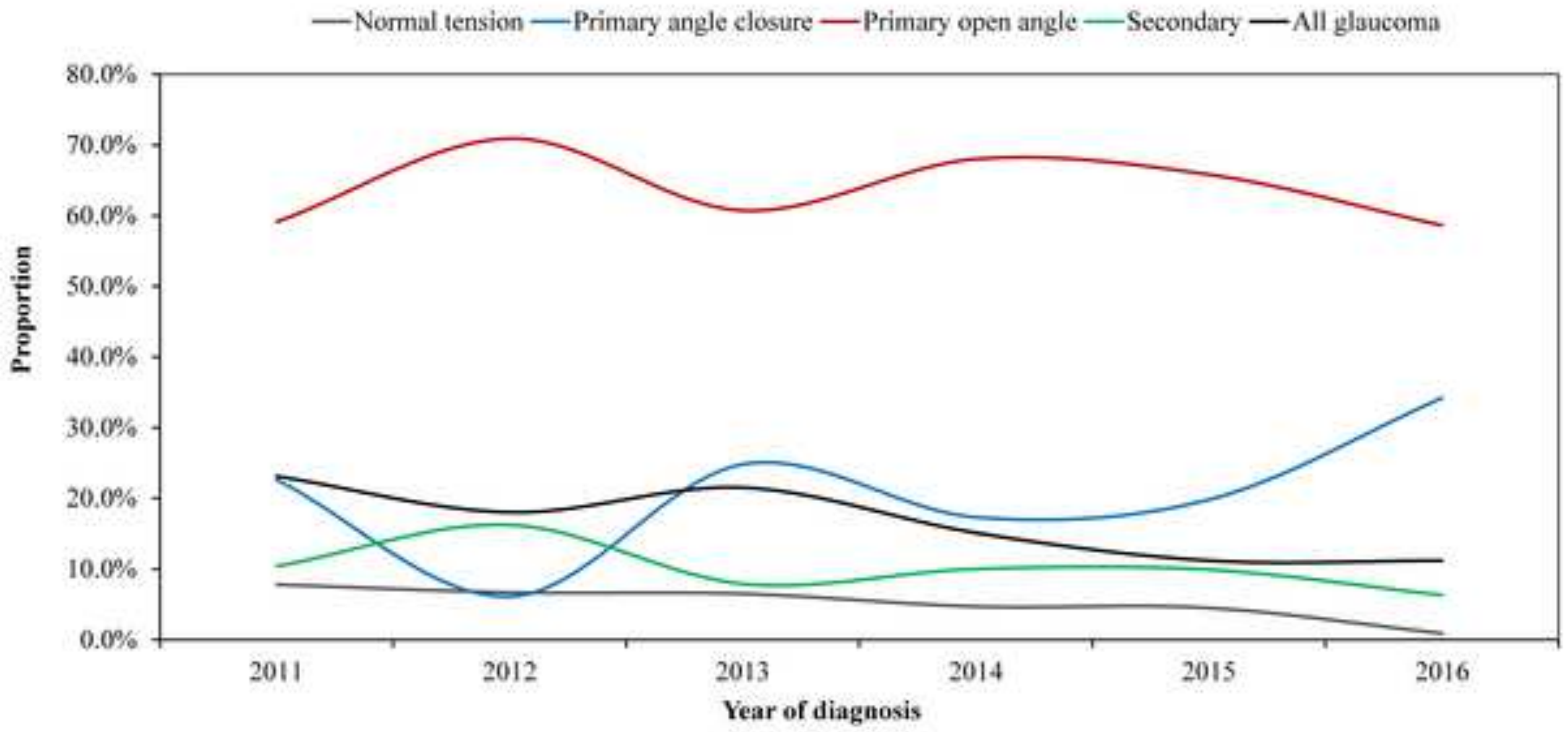

Figure 3

Click here to access/download;Figure;Fig 3.tif

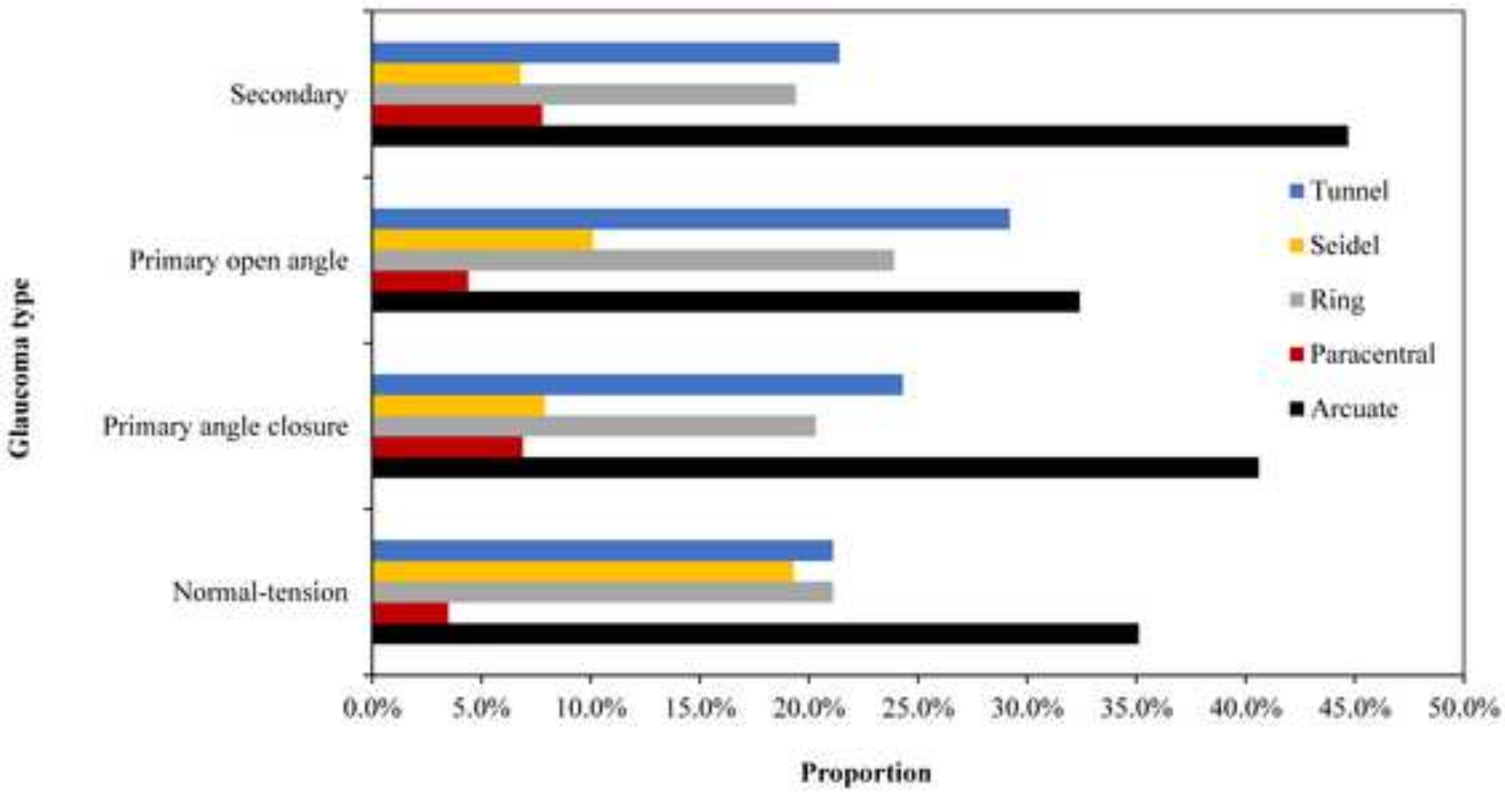

Supplement: Attachment — Submitted filename: PONE-D-21-21579 PLOS ONE.pdf [file pone.0260965.s002.pdf]
