# Supplementary material for: Prevalence and clinical profile of glaucoma patients in rural Nigeria—A hospital based study
Source: PLoS One. 2021 Dec 2;16(12):e0260965. doi: 10.1371/journal.pone.0260965 (PMC8639068; doi:10.1371/journal.pone.0260965)
Supplement: Attachment — Submitted filename: PONE review response.docx [file pone.0260965.s003.docx]

PONE-D-21-21579R1

Prevalence and clinical profile of glaucoma patients in rural Nigeria -  A hospital based study Dr Uchechukwu Levi Osuagwu

Dear Dr. Osuagwu,

We've checked your submission and before we can proceed, we need you to address the following issues:

1. Thank you for stating the following financial disclosure:

[Not applicable].

At this time, please address the following queries:

a) Please clarify the sources of funding (financial or material support) for your study. List the grants or organizations that supported your study, including funding received from your institution.
b) State what role the funders took in the study. If the funders had no role in your study, please state: “The funders had no role in study design, data collection and analysis, decision to publish, or preparation of the manuscript.”
c) If any authors received a salary from any of your funders, please state which authors and which funders.
e) If you did not receive any funding for this study, please state: “The authors received no specific funding for this work.”

Please include your amended statements within your cover letter; we will change the online submission form on your behalf.

Reply: Done. See cover letter and wording was revised in the manuscript to reflect this. Thanks for changing the online submission on our behalf

2. In the ethics statement in the manuscript and in the online submission form, please provide additional information about the patient records used in your retrospective study, including:

a) whether all data were fully anonymized before you accessed them;

Reply: This has been included in the manuscript.

“…..Only the researcher (CO) has access to identifiable participant data in order to extract the required variables. However, the researcher de-identified the data once the variables were extracted.

[line 116-118]”

b) the date range (month and year) during which patients' medical records were accessed.

Reply: This was already in the manuscript lines 100-101 and read: ….January 2011 and December 2016

If the ethics committee waived the need for informed consent, or patients provided informed written consent to have data from their medical records used in research, please include this information.

**Reply: Done. Line 201**

3. We note your updated Data Availability Statement: “All relevant data are within the manuscript and additional information can be obtained on reasonable request by the Corresponding author"

Please address the following:

a. We note that you state that all relevant data are within the manuscript. Please confirm at this time whether or not your submission contains all raw data required to replicate the results of your study. Authors must share the “minimal data set” for their submission. PLOS defines the minimal data set to consist of the data required to replicate all study findings reported in the article, as well as related metadata and methods (<https://journals.plos.org/plosone/s/data-availability#loc-minimal-data-set-definition>).

For example, authors should submit the following data:

- The values behind the means, standard deviations and other measures reported;

- The values used to build graphs;

- The points extracted from images for analysis.

Authors do not need to submit their entire data set if only a portion of the data was used in the reported study.

If your submission does not contain these data, please either upload them as Supporting Information files or deposit them to a stable, public repository and provide us with the relevant URLs, DOIs, or accession numbers. For a list of recommended repositories, please see <https://journals.plos.org/plosone/s/recommended-repositories>.

Reply: The data has been uploaded as supporting information file and revised the availability of data and material section.

**“Availability of data and material**: The data that support the findings of this study are included in the manuscript. In addition, the de-identified data required to replicate all study findings reported in the article are included as supplementary file”

b. We also note that additional data will be available from the corresponding author. PLOS only allows data to be available upon request if there are ethical, legal, or third-party restrictions on sharing a de-identified data set. Please also note that in the interest of long-term data availability, PLOS data policy states that it is not acceptable for an author to be the sole named individual responsible for ensuring data access. (<https://journals.plos.org/plosone/s/data-availability#loc-acceptable-data-access-restrictions>)

If there are restrictions, please explain them in detail (e.g., data contain potentially sensitive information, data are owned by a third-party organization, etc.) and who has imposed them (e.g., an ethics committee). Please also provide contact information for a data access committee, ethics committee, or other institutional body to which data requests may be sent. If data are owned by a third party, please indicate how others may request data access.

This information will be helpful in updating your Data Availability Statement. We look forward to hearing from you.

Reply: The statement has been revised and raw data that is required to replicate all results presented have been uploaded as supplementary file. I have also included it in the cover letter.

Kind regards,

Osuagwu Uchechukwu Levi

Corresponding Author
