# Supplementary material for: Prevalence and clinical profile of glaucoma patients in rural Nigeria—A hospital based study
Source: PLoS One. 2021 Dec 2;16(12):e0260965. doi: 10.1371/journal.pone.0260965 (PMC8639068; doi:10.1371/journal.pone.0260965)
Supplement: Attachment — Submitted filename: Responses to the reviewers for PLOS ONE journal.docx [file pone.0260965.s004.docx]

**Rebuttal letter: Response to reviewers’ comments**

Dear Editor, thanks for the comments. We had earlier addressed all the reviewers’ comments and those of the editor and we were surprised that you didn’t receive that even though it was in our attached submission. I have again attached the response file for your perusal and highlighted the sections that addressed the reviewers concerns. In addition, we have worked on the recent comment as shown below

**New file**

PONE-D-21-21579R1

Prevalence and clinical profile of glaucoma patients in rural Nigeria -  A hospital based study

PLOS ONE

Dear Dr. Osuagwu,

Thank you for submitting your manuscript to PLOS ONE. After careful consideration, we feel that it has merit but does not fully meet PLOS ONE’s publication criteria as it currently stands. Therefore, we invite you to submit a revised version of the manuscript that addresses the points raised during the review process.

==============================
ACADEMIC EDITOR:

I cannot find a point-to-point response to reviewers' comments, nor changes in the manuscript in response to these comments. The authors only addressed the changes needed by the journal, but not the reviewers.
==============================

Response: Thanks for the comment. We have addressed all comments in the manuscript and provided a point by point response to all comments in the attached rebuttal letter. It came as a surprise that the reviewer could not find that in the merged document. Below are the responses and all changes in the manuscript have been highlighted in different fonts

Please submit your revised manuscript by Dec 10 2021 11:59PM. If you will need more time than this to complete your revisions, please reply to this message or contact the journal office at [plosone@plos.org](mailto:plosone@plos.org). When you're ready to submit your revision, log on to <https://www.editorialmanager.com/pone/> and select the 'Submissions Needing Revision' folder to locate your manuscript file.

Please include the following items when submitting your revised manuscript:

- A rebuttal letter that responds to each point raised by the academic editor and reviewer(s). You should upload this letter as a separate file labeled 'Response to Reviewers'.

Done

- A marked-up copy of your manuscript that highlights changes made to the original version. You should upload this as a separate file labeled 'Revised Manuscript with Track Changes'.

Done

- An unmarked version of your revised paper without tracked changes. You should upload this as a separate file labeled 'Manuscript'.

Done

**Reviewers' comments:**

[NOTE: If reviewer comments were submitted as an attachment file, they will be attached to this email and accessible via the submission site. Please log into your account, locate the manuscript record, and check for the action link "View Attachments". If this link does not appear, there are no attachment files.]

While revising your submission, please upload your figure files to the Preflight Analysis and Conversion Engine (PACE) digital diagnostic tool, <https://pacev2.apexcovantage.com/>. PACE helps ensure that figures meet PLOS requirements. To use PACE, you must first register as a user. Registration is free. Then, login and navigate to the UPLOAD tab, where you will find detailed instructions on how to use the tool. If you encounter any issues or have any questions when using PACE, please email PLOS at [figures@plos.org](mailto:figures@plos.org). Please note that Supporting Information files do not need this step.

Response: Done

**Old file**

**Response to Reviewer #1 comments**:

This manuscript requires revision in order to make it useful to the reader.

Introduction.
Lines 81-4: An explanation is required as to how this study will shed light on inter-ethnic and regional variations in glaucoma prevalence in Nigeria, as the study was based in a particular hospital (single centre).

**Response**

Various studies [12-14] in different parts of Nigeria have shown that glaucoma is one of the leading causes of blindness in the country and the prevalence is slightly higher in the Southeastern part of the country compared with other regions. In a 1995 population based cross sectional survey conducted in Dambatta local government area (LGA), Kano state, Northwestern Nigeria, the authors reported that 15% of the blindness and 7% of the visual impairment they found, were attributable to glaucoma [15]. Murdoch et al [6] reviewed population based studies published between 1966 to September 2012 on posterior segment eye diseases (PSEDs) in sub-Saharan Africa. They found that in Nigeria, the prevalence of glaucoma was 1.02% in those aged >45 years and noted that African-based studies are needed to help estimate present and future needs and plan services to prevent avoidable blindness.

Nigeria is divided along three main ethnic groups with the Igbos in the Eastern region, Yorubas in the Western region and Hausas in the Northern region. Each ethnic group has its unique culture and the lack of ethnic specific data on sight-threatening diseases such as glaucoma makes it difficult to extrapolate the one group’s findings due to differences in cultural and socio-economic activities. There is a need to understand the demographic and clinical presentation of glaucoma in different regions in Nigeria for effective management. Evaluating the epidemiological and clinical profile of glaucoma patients seen at the Federal Medical Centre Eye clinic Gusau, Zamfara State will shed light on inter-ethnic and regional variations of glaucoma prevalence in Nigeria. It will also provide a useful background information for planning epidemiological surveys on glaucoma in this region as well as other parts of Nigeria with similar socio-demographic and ecological characteristics. Therefore, this study was aimed to assess the epidemiological characteristics and clinical presentations of glaucoma patients’ ≥50 years seen at a referral center in Nigeria. (Line 73-94).

**Reviewer’s comment**

Was the population multi-ethnic?

**Response**

No, the population was mostly Hausa because the study was done in the north where majority of the population are Hausas. However, there was still a good proportion of people from other ethnic groups. The relevant information from the study was mainly to highlight the prevalence of glaucoma among Hausa ethnic groups in this region so as to compare with the findings from other regions including studies done in the South East and South West where majority of the population are of Igbo and Yoruba ethnic groups respectively.

This was also highlighted in the result. The relevant section reads: Table 1 presents the characteristics of this study population indicating that nearly all were Muslims, females (56.7%) and of Hausa origin.

**Reviewer’s comment**
Methods. The authors need to state how cases of glaucoma were identified: was there a database, registry, or coding system that allowed such identification, other system employed?

**Response**

Done. This has been added to the manuscript for clarification (Line 123-129).

The hospital does not have electronic records, coding, and database registry. The hospital still operates in hard copy system for storing patients’ records’ therefore all case files of adult patients diagnosed with glaucoma from 2011 to 2016 were first requested from the hospital administrator and retrieved from the archives with the assistance of the eye clinic department secretary. Data collection involved the use of a data extraction sheet to extract information on demographics, and clinical profile directly from the patients’ files.

**Reviewer’s comment**
Was VA measured in logMAR directly or in Snellen and subsequently converted? See later as well (lines 76=9).

**Response**

All VAs were measured in Snellen and subsequently converted to logMAR. It was previously explained in the old version as stated below:

for purposes of analysis, participants with counting finger at 2 feet were considered to have a visual acuity of 2/200 or 20/2000. Those with hand movement at a distance of 2 feet were considered to have an equivalent Snellen acuity of 20/20,000. Also, these were converted to logMAR. Light perception (LP) with or without projection and no light perception (NLP) are not VA measurements but merely the ability to detect a stimulus (Line 187-192).

**Reviewers comment**

What type of prevalence was this, and how was it determined (lines 221-4)? As the authors are aware, hospital prevalence is totally different from population prevalence, and have different implications.

**Response**

The prevalence of glaucoma reported in this study was a hospital prevalence as only those who attended the hospital and were diagnosed with glaucoma participated in the study. We also made this clear in the previous version of the manuscript (line 113-115) as stated below:

### Data for all participants aged 50 years and over who presented for the first time to this referral center and were diagnosed with glaucoma at the eye clinic during the study period were included.

### We have also made it clearer in the subtitle: Hospital Prevalence of Glaucoma

**Reviewer’s comment**
How was the changing frequency of different subtypes of glaucoma determined?

**Response**

The changing in frequency of different subtypes of glaucoma was gotten by calculating the total number of people with a particular glaucoma type divided by the total number with glaucoma in that year multiplied by 100. It has also been added to the manuscript (184-186).

**Reviewer’s comment**

How do the authors explain the falling frequency of glaucoma over time?

**Response**

The study found a marked reduction in the prevalence of all other glaucoma types including POAG, which might not necessarily reflect reduction in glaucoma prevalence but rather a decrease in the utilization of eye care services triggered by insurgency and civil unrest predominant in this region [18]. In addition to these factors, poor awareness of glaucoma and low life expectancy in Nigeria could play a role in the decline in glaucoma prevalence [18, 43]. This was previously explained in the old version of the manuscript (line 316-321).

**Reviewer’s comment**
The Discussion could be slightly focused.

**Response**

The discussion has been revised to be more focused.

**Reviewer’s comment**
Other comments
Line 44, 123.202 and elsewhere: 'hemi-field'

**Response**

All hemi-field have been corrected to hemifield

**Reviewer’s comment**
Line 45 and elsewhere: p value with lower case 'p'

**Response**

All p value have been changed to *P*

**Reviewer’s comment**
Line 576: insert 'and/or' preceding 'irreversible'

**Response**

The revision has been added in the manuscript (Line 57).

**Reviewer’s comment**
Line 111: replace ';' with stop, and commence a new sentence to read 'Further, those with a history....'

**Response**

The semicolon has been replaced with a full stop (Line 117).

**Reviewer’s comment**
Line 120: define 'IOP' at first use in the manuscript (I cant find it)

**Response**

IOP has been defined appropriately at first use as intraocular pressure (IOP) in the manuscript (Line 131).

**Reviewer’s comments**
Line 136: 'VCDR previously defined in line 120; adopt abbreviation subsequently throughout.

**Response**

VCDR abbreviation has been modified throughout.

**Reviewer’s comment**
Line 174: 'logMAR'

**Response**

LogMar has been changed to logMAR in the manuscript.

**Reviewer’s comment**
Lines 76-9: define these levels of VA in logMAR. Similarly HM, CF and PL have logMAR notations?

**Response**

The section has been revised and the levels of VA has been explained.

Similar to previous paper [25] and for purposes of analysis, participants with counting finger at 2 feet were considered to have a visual acuity of 2/200 or 20/2000. Those with hand movement at a distance of 2 feet were considered to have an equivalent Snellen acuity of 20/20,000. Also, these were converted to logMAR. Light perception (LP) with or without projection and no light perception (NLP) are not VA measurements but merely the ability to detect a stimulus. Therefore, these factors were excluded from the analysis [26].

**Reviewer’s comment**

**Response to Reviewer #2 comments**

**Reviewer’s comment**

The authors are not clear with their unit of analysis. they reported that 995 patients with glaucoma were diagnosed over the 5 years of their study and they proceeded to present field defects of 995 patients however they didn't mention which one of the patient was selected for analysis and what criteria was used in the selection of the eye.

**Response**

All the 995 participants with glaucoma were included in the analysis and no participant was excluded.

**Reviewer’s comments**

Furthermore, they stated in line 209 to 211 that 15 patients had visual acuity of light perception in both eyes and that these patients were excluded from analysis (line 177 to 179) then how did they arrive at 995 visual defect results when 15 of the 995 had only light perception bilaterally.

**Response**

No participant was excluded in the analysis as that was written in error.

**Reviewer’s comment**

Additionally, it is impossible for HVF machine to report GHT results without reporting the global indices.

**Response**

It is true that Humphrey visual field test report GHT and global indices. However, in this hospital it is not common practice for ophthalmologists to record global indices in patients’ files. So we recorded only data found in patients’ files. This limitation has also been included in the manuscript (Line 386-387).

**Reviewer’s comment**

As a glaucoma specialist who has been treating glaucoma patients in Nigeria, I know it is almost impossible to see only 995 glaucoma patients in 5 years.

**Response**

We have now indicated that this was a hospital prevalence and we agree with the reviewer that it might not be the true reflection of prevalence of glaucoma in Nigeria. However, the prevalence of glaucoma recorded was based on the record given to us by the hospital.

**Reviewer’s comment**

The glaucoma definition the author's quoted in the study is applicable only in prevalent survey not for hospital based study.

**Response**

The glaucoma definition used was based on the information obtained from the hospital which was confirmed in other studies (line 144-145).
